# Belowground plant development measured with magnetic resonance imaging (MRI): exploiting the potential for non-invasive trait quantification using sugar beet as a proxy

**DOI:** 10.3389/fpls.2014.00469

**Published:** 2014-09-16

**Authors:** Ralf Metzner, Dagmar van Dusschoten, Jonas Bühler, Ulrich Schurr, Siegfried Jahnke

**Affiliations:** Institute of Bio- and Geosciences, IBG-2: Plant Sciences, Forschungszentrum Jülich GmbHJülich, Germany

**Keywords:** *Beta vulgaris* ssp. *vulgaris* (sugar beet), cambial rings, imaging (3D), magnetic resonance imaging (MRI), non-invasive method, root development

## Abstract

Both structural and functional properties of belowground plant organs are critical for the development and yield of plants but, compared to the shoot, much more difficult to observe due to soil opacity. Many processes concerning the belowground plant performance are not fully understood, in particular spatial and temporal dynamics and their interrelation with environmental factors. We used Magnetic Resonance Imaging (MRI) as a noninvasive method to evaluate which traits can be measured when a complex plant organ is monitored *in-vivo* while growing in the soil. We chose sugar beet (*Beta vulgaris* ssp. vulgaris) as a model system. The beet consists mainly of root tissues, is rather complex regarding tissue structure and responses to environmental factors, and thereby a good object to test the applicability of MRI for 3D phenotyping approaches. Over a time period of up to 3 months, traits such as beet morphology or anatomy were followed in the soil and the effect of differently sized pots on beet fresh weight calculated from MRI data was studied. There was a clear positive correlation between the pot size and the increase in fresh weight of a sugar beet over time. Since knowledge of the development of internal beet structures with several concentric cambia, vascular and parenchyma rings is still limited, we consecutively acquired 3D volumetric images on individual plants using the MRI contrast parameter T_2_ to map the development of rings at the tissue level. This demonstrates that MRI provides versatile protocols to non-invasively measure plant traits in the soil. It opens new avenues to investigate belowground plant performance under adverse environmental conditions such as drought, nutrient shortage, or soil compaction to seek for traits of belowground organs making plants more resilient to stress.

## Introduction

Roots of vascular plants are specialized in mechanical anchoring and resource acquisition often combined with nutrient storage, all of which are key processes for plant performance and crop yield (Waisel et al., 2002; Gregory, 2006). Nevertheless, our knowledge of the development of root structure and function is lagging behind that of the shoot mainly due to the opacity of the soil, hindering direct observation. Roots are also sensitive to excavation, which further hampers studying intact and functional root systems (Gregory, 2006) as well as other belowground structures such as storage organs and their development. Another hindrance when studying roots is that they develop naturally in a 3D soil environment with biotic and abiotic factors (such as water, nutrients, mycorrhiza, or compaction zones) much less homogenously distributed than for shoots in the airspace. Roots have adapted to this by developing in a flexible 3D pattern while exploring their soil environment and interacting with it, leading to strong differences between the root systems even of plants of the same genotype (Gregory, 2006; Eshel, 2013). While the architecture of the root system can be complex, the anatomy of roots may be relatively simple. On the other hand the anatomy of belowground storage organs can be rather complicated in particular when supernumerary or anomalous cambia come into play as for sugar beet (Artschwager, 1926) or sweet potato (Villordon et al., 2009), and also the resulting morphology may be important for their function, yield and economic value. Sugar beet has been investigated here as a proxy for a very complex belowground plant structure, since it consists of tissues originating from the stem, the hypocotyl and to the largest extent, the root (Artschwager, 1926; Draycott, 2006). During growth, all traits of a beet may be affected by adverse circumstances like obstacles present or wounding with obvious growth distortions, giving visible indication of suboptimal growth conditions (Draycott, 2006). Often more than a dozen concentric cambial rings, which are simultaneously active (Artschwager, 1926), are responsible for the anomalous secondary thickening of the beet, and they are also tightly linked to the sugar storage function since each cambium ring produces both transport and storage tissues that form the body of the beet (Artschwager, 1926; Fieuw and Willenbrink, 1990; Draycott, 2006). Morphological traits of the beet may be responsible for adhering soil at harvest that needs to be removed before processing (Elliot and Weston, 1993), which accounts for additional costs, and different morphological sections of a belowground organ like sugar beet may differ in economic value (Mahn et al., 2002). Sugar beet (*Beta vulgaris* ssp. *vulgaris* L.), is an important crop contributing to about one quarter of global sugar production of 160 Mt (Biancardi et al., 2010). Modern cultivars of sugar beet accumulate sucrose up to 20% of the beet fresh weight (Draycott, 2006) but, as valid for all crops, there is need for continued research either to keep performance under less than optimal conditions or to increase performance. Destructive excavation studies delivered mainly snapshots of the different growth stages (Artschwager, 1926, 1930; Rapoport and Loomis, 1986; Hoffmann, 2010). Non-invasive approaches with rhizotrons (Gregory, 2006; Neumann et al., 2009) are not well suited to study solid volumetric (3D) structures or gain anatomical information on ring development. Linking structure and function and correlating them to yield or yield stability has proven to be difficult (Doney et al., 1981; Hoffmann, 2010), possibly due to the lack of detailed developmental data.

Two methods are currently used for non-invasive 3D imaging of roots growing in soil: X-ray computed tomography (CT) and magnetic resonance imaging (MRI). CT is more widely used due to lower costs of the instruments and the high spatial resolution it delivers (Mooney et al., 2012). The contrast is based on X-ray attenuation, which provides only low contrast both between different plant tissues (Han et al., 2008; Jung et al., 2012) and between plant roots and soil (Gregory et al., 2003; Mooney et al., 2012), thus requiring highly advanced algorithms for automated segmentation of root structures (Mooney et al., 2012). MRI on the other hand offers a wide range of contrast parameters for segmentation of organs and tissue structures. The basic principles of MRI and its use in biomedical sciences are described in detail in several textbooks (Callaghan, 1993; Haacke et al., 1999) or articles focusing on applications in plant biology (e.g., Köckenberger et al., 2004; Blümler et al., 2009; Van As et al., 2009; Borisjuk et al., 2012). In plant sciences, applications range from imaging of fruits, seeds, roots or shoot structures (Fedotov et al., 1969; Brown et al., 1986; Kuchenbrod et al., 1995; Köckenberger et al., 2004; Van As et al., 2009; Borisjuk et al., 2012) to measuring water status or water flow in plant organs (Köckenberger, 2001; Windt et al., 2006). Pioneering work on storage organs has been conducted on sugar beet (Kano et al., 1993; Macfall and Johnson, 1994) and *Zantedeschia* tuber (Robinson et al., 2000), which both were excavated prior to MRI measurements. Here, we employed MRI to monitor belowground storage organs of sugar beet in pots up to 117 mm inner diameter for which appropriate MRI instruments and protocols had to be established. A brief overview of the method will be given including specific challenges for MRI imaging of storage roots growing in soil.

MRI is based on nuclear magnetic resonance (NMR), which exploits an intrinsic angular movement of atomic nuclei, called spin, lending some of them like ^1^H (protons) a weak magnetic moment. ^1^H is the most often used nucleus in MRI due to both high detection sensitivity and abundant presence in living tissues. The imaging contrast in plant tissues is, beside the differences in proton density, most notably determined by the time constant of the signal decay. It is also called “relaxation of nuclear magnetization” and has a longitudinal (T_1_) and a transverse (T_2_) component with respect to the external magnetic field. Depending on the measurement settings, images can be produced that are mainly contrasted by either proton density, T_1_, T_2_ or any mixture of these three. This can be exploited to obtain optimal visibility of the targeted structures. The presence of ferromagnetic particles and other soil properties (such as particle size composition) may have negative effects on image quality (Rogers and Bottomley, 1987; Asseng et al., 2000) but, when substrates are carefully selected for low content of ferromagnetic particles or freed of the strongest ferromagnetic particles, MRI can provide rather good images of roots growing in soil (Bottomley et al., 1986; Rascher et al., 2011).

First MRI images of sugar beet in the soil were to our knowledge shown in a study exploring the usability of combined MRI-PET (positron emission tomography) measurements on different plant species (Jahnke et al., 2009). Studying the development of sugar beet requires clear contrast against the surrounding soil, detecting 3D beet morphology, estimation of fresh weight and visualization of the ring structures. Our goal was to test which traits, relevant for development and yield of belowground storage organs, could be monitored with specifically designed MRI instrumentation and protocols. For sugar beets growing in pots filled with soil, we acquired data that allowed quantification of beet morphology, tissue structures and their development as well as pot size effects on the fresh weight of the investigated beets.

## Materials and methods

### Plant material, substrate and pots

Sugar beet plants (*Beta vulgaris* ssp. vulgaris var. altissima L.; cultivar “Pauletta,” KWS, Einbeck, Germany) were grown from seed in a growth chamber in a mixture of homogenized agricultural topsoil and coarse sand (1:2; v/v). This mix is suited for MRI application (Rascher et al., 2011; Hillnhütter et al., 2012) but was not tested yet for sugar beet cultivation. The agricultural soil, characterized as a gleyic cambisol, was collected by removing the top 30 cm from a farmer's field (Kaldenkirchen, Germany) and air dried. Subsequently, the soil was powdered and homogenized in a drum hoop mixer (J. Engelsmann, Ludwigshafen, Germany), sieved to 2 mm and freed of stronger ferromagnetic particles by moving it in a thin layer on a conveyor belt through a perpendicular magnetic field provided by rare earth magnets (NdFeB N42, 1.3 T; Webcraft GmbH, Gottmardingen, Germany). Coarse quartz sand (grain size 0.71–1.4 mm; Quartzwerke Witterschlick, Alfter, Germany) was similarly freed of ferromagnetic particles. In total, substrate preparation took about 8 min per liter. The ready mixture was filled into PVC tubes of two different sizes with (a) an inner diameter (I.D.) of 81 mm, a height of 400 mm and a volume of 2.1 L and (b) an I.D. of 117 mm, a height of 800 mm and a volume of 8.7 L. Both had 8 mm holes in the bottom caps for drainage and aeration covered with nylon mesh (grid size 200 μm) to prevent loss of substrate and roots growing out.

### Plant cultivation

The pots were watered to above container capacity and, after excess water had drained away, three seeds were laid down in holes 2 cm deep and covered with soil. After germination (5–8 days after sowing, DAS), the pots were watered automatically once per day with a nutrient solution (0.01% Hakaphos blue; Compo, Münster, Germany) that was increased stepwise to 5 times per day until week 8 after sowing. The nutrient concentration was raised after 3 weeks to 0.03% and after 8 weeks to 0.05%. The growth chamber was set to 16:8 h, light: dark and 20:16°C, respectively, while relative humidity was kept constant at 60 ± 3%. Lighting was provided by 5 × 400 W HPI and 5 × 400 W SON-T lamps (both Philips, Hamburg, Germany) that alternated every 2 h with 5 min overlap giving PAR intensity between 350 and 450 μmol m^−2^ s^−1^ at canopy level.

### Harvest and photographs

Immediately after the respective last MRI measurement, plants were photographed with a digital camera (D 70; Nikon, Tokyo, Japan) and removed from the pots. Beets were washed carefully with tap water to remove adhering soil while keeping side roots intact and dried with paper towels. Afterwards the whole plants were again photographed from the same perspective as before. Fresh weight was taken after removal of the leaves and unthickened roots. Selected beets were sectioned by hand and stained with Astra blue and Safranine, and light micrographs were taken using an Axioplan/Axiophot 2 microscope (Zeiss, Oberkochen, Germany) with attached Nikon D3 Camera at ×50 magnification.

### MRI instrument and measurement setup

MRI measurements were performed on a plant dedicated vertical bore 4.7 T magnet equipped with gradient coils providing 300 mT m^−1^ (Varian, Palo Alto, USA). For plants grown in 81 mm I.D. pots, we used a 100 mm I.D. RF coil (sensitive vertical length 100 mm; Varian, Palo Alto, USA). For 117 mm pots, we used a 170 mm I.D. RF coil (sensitive vertical length 120 mm; RAPID Biomedical, Würzburg, Germany). Experimental control was run on a Varian VNMRS console and a Linux PC using the Varian software VnmrJ. During measurements the plants were positioned in the bore of the magnet at a temperature of 18°C. For 3D images, a 3D spin echo sequence was used (single echo) that subdivided a selected region into a 3D voxel grid (voxel = volumetric pixel) and required a 3D Fourier transformation for image reconstruction (Haacke et al., 1999). Repetition time (T_*R*_) was set to 200 ms and echo time (T_*E*_) was set to 12 ms. Each measurement took about 10 min. In case additional T_2_ maps were acquired, a multi spin echo sequence (*T*_*R*_ = 1200 ms, *T*_*E*_ = 5.4 + n^*^5.4 ms, *n* = 0 … 7) was used giving multiple echoes for 5 slices positioned horizontally through the thickest part of the beet and spaced 4 mm apart. The sequence was custom written and, by keeping echo times short, negative effects of diffusion through background gradients (caused by air pockets inside the beet) were minimized (Edzes et al., 1998). Measurement time for T_2_ maps with a field of view of 63 × 63 mm^2^ and pixel size of 164 × 164 μm^2^ was about 30 min.

### Data handling, image processing and biomass calculation

For image visualization and 3D representations of the datasets, the software package Mevislab (version 2.2.1, MeVis Medical Solutions, Bremen, Germany) was used in combination with Matlab (Mathworks, Natick, USA) and the open source Matlab toolbox AEDES (version r172, www.aedes.uef.fi). The 3D MRI datasets of the sugar beets were processed with Mevislab where the beet volumes were segmented from noise, water in soil pockets, petioles and the unthickened taproot by manually setting an intensity threshold and a region of interest under visual control. From a segmented beet the maximal beet diameter was automatically measured for all virtual axial slices using a home written Matlab script. Here, the maximal beet diameter was defined as the maximal possible distance line covering the centroid for all virtual axial slices. For the eight plants grown in 81 mm pots, the mean of the maximum diameters is shown in Table 1.

**Table 1:** **Development of the maximal diameter and volume of eight sugar beet plants grown in 81 mm ID pots in a climate chamber, including the specimen shown in Figure 2, given as arithmetic mean ± *SD***.

**DAS [days]**	**Diameter [cm]**	**Volume [cm^**3**^]**
53	1.5 ± 0.3	5.5 ± 2.2
67	2.5 ± 0.4	15.3 ± 4.8
81	3.3 ± 0.4	31.0 ± 7.1
102	4.4 ± 0.3	57.4 ± 9.2
130	5.0 ± 0.4	104.0 ± 9.9

DAS, days after sowing.

For an undisturbed beet plant growing in soil, a “calculated fresh weight” (cFW) was obtained by multiplying the beet volume measured with MRI with a density derived from different beets of the same cultivar grown under similar conditions. For these reference plants the beet volume was measured with MRI, and directly afterwards the respective fresh weight (FW) of the harvested beet was determined resulting in a mean density of 1.17 ± 0.16 g ml^−1^ (mean ± *SD*; *n* = 21 plants). Calculated fresh weight for each pot size is displayed in **Figure 3** as mean ± *SD* (*n* = 8) along with a polynome of 2nd order fitted to the mean values (*R*^2^ = 0.9998) with Sigma Plot (version 11, Systat Software, San Jose, USA). This fit was also used to extrapolate values for the cFW-81 curve in **Figure 3** beyond 129 DAS. For comparing the development of beet biomass we first plotted the individual data points from both pot sizes (cFW-81 and cFW-117) against time and fitted quadratic regressions separately for each pot size and for all data in Sigma Plot. Then we performed a sum of squares reduction test (Gallant, 1987) using Excel (version 14.0, Microsoft, Redmont, USA) to evaluate significance of pot size effects. The functions of those fits were used to interpolate the values for cFW-81 on 53 DAS and cFW-117 (Table 2) on 118 DAS respectively.

**Table 2:** **Belowground fresh weight (mean ± *SD*; *n* = 8 plants) and fresh weight ratios of beets grown in pots of two different sizes, based on the same dataset as presented in Figure 3**.

**Pot inner diameter [mm]**	**Pot Volume [L]**	**cFW 53 DAS [g]**	**cFW 118 DAS [g]**	**Ratio cFW DAS118 / DAS53**
81	2.1	6.5 ± 2.6	83.6 ± 10.8^*^	12.9
117	8.7	10.8 ± 4.0^*^	155.9 ± 17.2	14.5

The numbers marked by an asterisk (

* ) were calculated from a 2nd order polynomial fitted to the data. cFW, calculated fresh weight; DAS, days after sowing.

### T_2_-maps and analyzing the width of beet rings

T_2_ relaxation times are correlated to characteristics of different plant tissues such as cell size, membrane permeability and solute content (Edzes et al., 1998; Van As et al., 2009) and may therefore be used for tissue identification (see Supplementary Information 1 for details). As we were focusing on the contrast between different tissues within the same image, we show here maps of T_2_ values. These were calculated from the MRI Data by fitting an exponential function to the multiple echoes, using the software IDL (ITT, Boulder, USA), which generated a proton density map at *t* = 0 and a T_2_ map, as described in detail by Donker et al. (1997) and Edzes et al. (1998).

On the T_2_-maps, ring widths were measured by setting seed points manually along the cambia that were automatically connected by closed splines using the Mevislab module *CSOFreehandProcessor*. The module *CSODistance* was used to calculate the mean distance between the segmented structures based on the minimal distance to the outer ring for each pixel of the inner ring.

## Results

### Belowground imaging of beet morphology and sugar beet development

**Figure 1:**
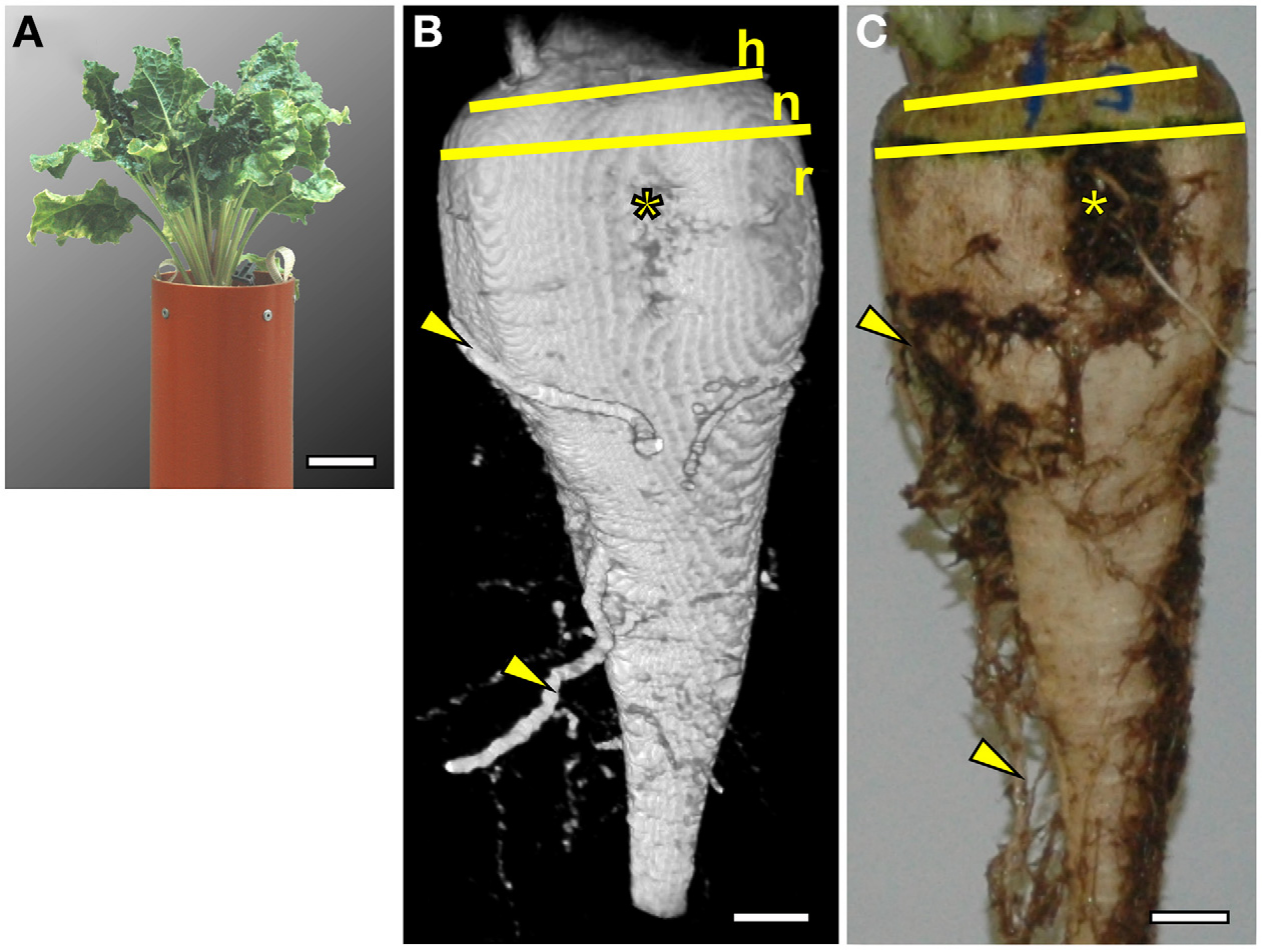
**Comparison of a sugar beet plant (*Beta vulgaris* ssp. *vulgaris* cv. “Pauletta”) photographed before and after harvest and imaged with Magnetic Resonance Imaging (MRI)**. **(A)** Photograph of the plant 118 days after sowing (DAS) growing in a soil-filled container with an inner diameter (I.D.) of 117 mm and a total height of 800 mm (only the upper part shown). **(B)** Volume rendering of MRI data showing both the outer shape of the beet and side roots in the soil. Different parts of the beet are indicated by yellow lines: the head of the beet (h) with the onset of petioles, the transitions zone of the neck (n), and the root part (r). A groove with rootlets (^*^) and some side roots (arrowheads) were visible as in **(C)**, an optical image of the same beet taken after excavation. The MRI image was obtained with a three dimensional spin echo sequence. The image size was 128 × 64 × 256 voxels (256 = vertical direction) with a field of view of 70 × 60 × 140 mm^3^ resulting in a voxel resolution of 0.54 × 0.94 × 0.54 mm^3^. Signal loss toward the top and bottom of the beet was caused by a loss of radio frequency (RF) homogeneity toward the upper and lower end of the RF coil. Scale bars: **(A)** 50 mm; **(B,C)** 10 mm.

In the substrate and containers described in the Materials and Methods section the sugar beet plants grew well (Figure 1A). Both the MRI image and the photograph after excavation (Figures 1B,C) showed a typically cone shaped beet (Artschwager, 1926) without growth distortions such as deformity or branching occurring under unfavorable conditions (Heinisch, 1960). The images are representative for a set of 8 plants grown under the same conditions (data not shown). With the applied measurement protocols, soil water gave a signal nearly two orders of magnitude lower than the water in the beet. The resulting high contrast allowed removal of the soil water signal by using a threshold cutoff filter without losing signal from the beet. Even part of the side roots with estimated diameters between 0.5 and 2 mm remained visible. In Figure 1B, the large side roots were in their native (3D) position in the soil whereas, after excavating and washing, the original alignment was lost (Figure 1C). Morphological details like the vertical groove, a typical trait of the root part of a sugar beet, were visible and, above the hypocotyl-derived neck, the stem-equivalent head could be recognized by insertions of the petioles (Figure 1B).

**Figure 2:**
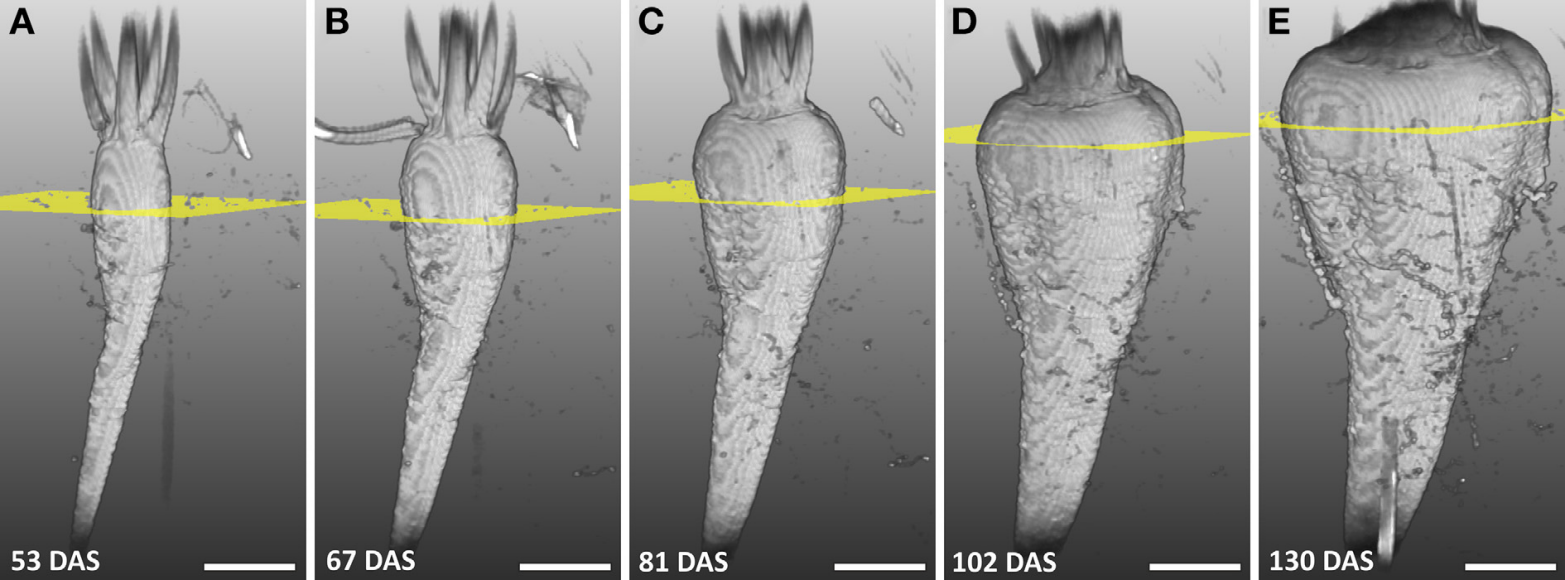
**Series of MRI images showing the development of a sugar beet in the soil with largest diameter highlighted**. **(A–E)** Volume renderings of 3D volumetric MRI datasets between 53 and 130 DAS. The sugar beet plant was grown and measured in a container with 81 mm I.D. and a height of 400 mm. Yellow planes denote the position of the largest diameter of the beet. In **(A–D)** the field of view (FOV) was 70 × 50 × 140 mm^3^ with an image size of 128 × 48 × 256 voxels and, in **(E)** the FOV was 70 × 70 × 140 mm^2^ with an image size of 128 × 64 × 256 voxels. The same measurement protocol as in Figure 1B was used. Scale bar: 20 mm.

The development of a sugar beet and its morphology was studied between 53 and 130 DAS by repeated imaging (Figure 2) showing a continuous increase in diameter and length of the beet together with a change in morphology. Until 81 DAS the thickest part was in the anatomical region of the root (Figures 2A–C) whereas, at 102 and 130 DAS, it was found in the neck section, which also grew in height (Figures 2D,E). The groove on the right side of the beet developed from barely visible at 48 DAS to a clear indentation in the circular beet circumference at 130 DAS (Figures 2A–E). Within the same time period, rootlets in the groove and side roots developed. The eight plants of the study showed similar development in morphology as the one of Figure 2 between DAS 53 and 130, resulting in an increase of the maximal beet diameter by more than three times from roughly 1.5–5 cm while the volume increased from about 6 cm^3^ to about 104 cm^3^ (Table 1). A video of the 3D growth of one of the beets from this series is shown in Supplementary Movie 1.

### Fresh weight development of beets growing in containers of two different sizes

**Figure 3:**
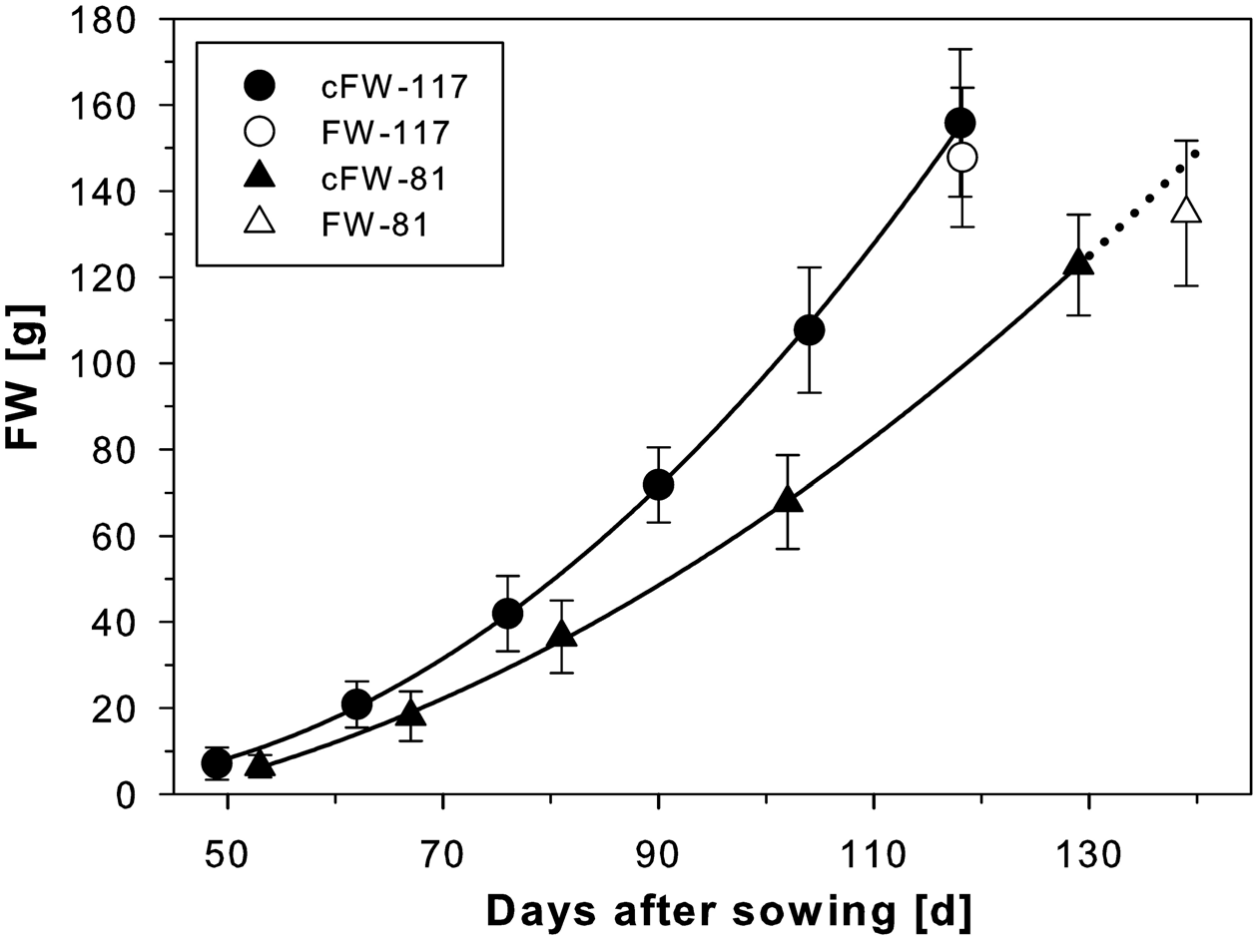
**Development of sugar beets grown in containers with either 117 mm I.D. (800 mm in height; cf. Figure 1) or 81 mm I.D. (400 mm in height; cf. Figure 2) for which calculated fresh weights of the beets (cFW-117 and cFW-81) were derived from MRI measurements every second week**. A polynomial of 2nd order was fitted to the data points (for both *R*^2^ = 0.9998). The fresh weight of the beets in the large containers was taken after harvest following the respective last MRI measurement at 118 DAS (FW-117). For the small containers, the cFW-81 curve beyond 129 DAS (dotted line) was extrapolated based on the polynomial for comparison with the fresh weight taken at slightly delayed harvest on 139 DAS (FW-81). The MRI measurements used the same MRI sequences and parameters as of Figure 1B. The values and error bars are given as arithmetic mean ± *SD* (*n* = 8 plants, respectively).

For two groups of beets grown in containers of different sizes under identical environmental conditions (Figure 3), the beet volumes were non-invasively measured over time with MRI and the beet fresh weights were calculated. The sugar beet plants grown in 117 mm I.D. pots showed higher cFW values than those grown in 81 mm I.D. pots (Figure 3) over the whole measurement period. To evaluate the significance of this pot size effect, we performed a sum of squares reduction test with the null hypothesis that there was no difference between plants growing in different pot sizes. The F-ratio turned out to be very high, *F*_*R*_ = 80.3 leading to an extremely low *p* value, *p* < 0.0001. Thus, the null hypothesis was rejected, proving that there was a statistically significant difference between the two groups of sugar beet plants over the time of observation. Between DAS 53 and 118, the cFW values of the beets increased by a factor of 12.9 in the small (I.D. 81) pots and a factor of 14.5 in the large (I.D. 117) pots (Table 2). The beet biomass in the large (8.7 L) pots was 66% and 86% higher than in the small (2.1 L) pots at DAS 53 and DAS 118, respectively. Poorter et al. (2012a, see Appendix 3 therein) observed that plant biomass generally scales with pot volume as *f*_*B*_ = *f*^*s*^_*v*_ where *f*_*B*_ = *B*_2_/*B*_1_ is the fraction by which biomass increases if pot size is increased by a factor *f*_*V*_ = *V*_2_/*V*_1_; the slope S can be determined as *S* = log(*f*_*B*_)/log(*f*_*V*_). For a doubling of the pot volume, i.e., *f*_*V*_ = 2, the increase in biomass is then *f*_*B*_ = 2^*S*^. Using this equation we calculated that the biomass of a double sized pot (4.2 L) compared to the small one (2.1 L) would have been 28 and 36% higher at DAS 53 and DAS 118, respectively.

### *In vivo* analysis of the beet anatomy at tissue level

**Figure 4:**
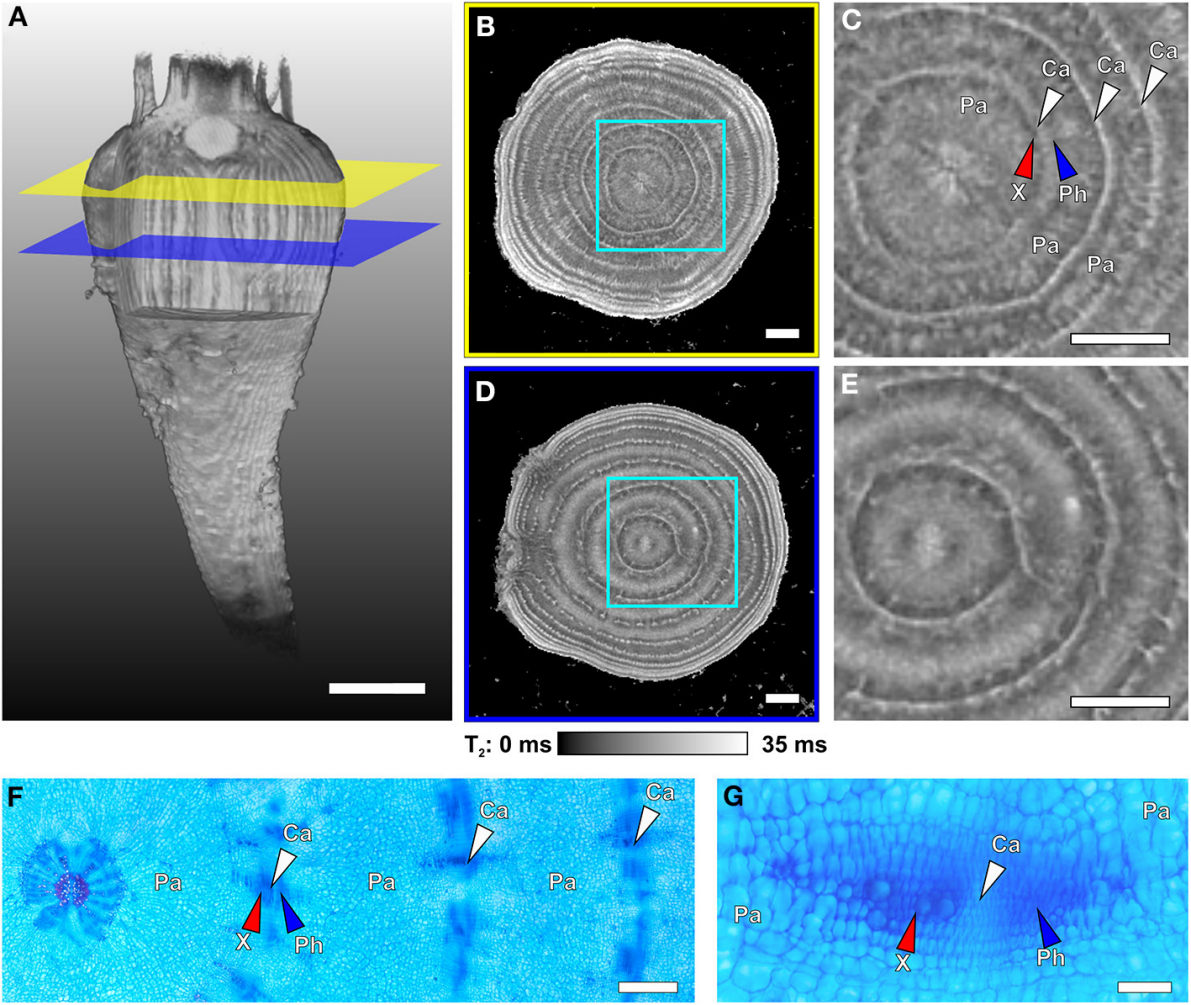
**Internal structures of different regions of a sugar beet imaged with MRI at 129 DAS and light microscopic images of cross sections taken from another plant of similar age**. **(A)** Volume rendering of the 3D dataset of the whole beet, with virtual cuttings to visualize internal longitudinal structures. Colored planes (yellow and blue) denote the positions of the virtual cross sections of **(B,D)**. Magnified sections of **(B,D)** are shown in **(C,E)**, respectively. The cross sections represent maps of the MRI contrast parameter T_2_ (transverse relaxation time) on virtual slices through the beet scaled from 0 to 35 ms; for clarity of view, T_2_ times >35 ms were set to 35 ms. For identification of the structures indicated by arrows in the MRI images they were highlighted in the microscopic images (stained with Astra Blue and Safranin), with **(F)** showing an overview including the beet core and three rings and **(G)** displaying higher magnification of a typical vascular bundle of such cambial rings. Identified tissues include parenchyma (Pa), cambium (Ca and white arrowheads), xylem (X and red arrowheads), and phloem (Ph and blue arrowheads). Plants were grown in tubes with 81 mm inner diameter. Image **(A)** was acquired with the MRI protocol of Figure 1B but with slightly modified parameters (FOV 70 × 70 × 140 mm^3^ with an image size of 128 × 64 × 256 voxels). The T_2_ maps of **(B,D)** were achieved with a multi-echo sequence (slice thickness in vertical direction 1 mm, FOV in plane 63 × 63 mm^3^ with an image size of 384 × 384 pixels). Scale bars: **(A–E)** 5 mm; **(F)** 1 mm; **(G)** 0.1 mm.

In Figure 4A the positions of two virtual slices through the thickest part of a sugar beet are indicated by intersecting planes. An MRI multi-echo multi-slice sequence was used to map the contrast parameter T_2_ of both slices at the same time, resulting in T_2_ maps (Figures 4B,D). Magnifications of the regions marked by cyan frames are shown in Figures 4C,E. The T_2_ maps of Figures 4B–E show concentric rings, which are narrow and bright indicating longer T_2_ values, alternating with darker rings of inhomogeneous gray values with shorter T_2_ times. Microscopic investigations on different beets harvested at the same age and developmental stage revealed the narrow bright rings in the MRI images as cambia (white arrowheads; Figures 4F,G). The bordering darker zones were identified as xylem and phloem (red and blue arrowheads respectively; Figures 4C,F,G) and the broad light gray rings between the cambial/vascular zones as parenchyma (Pa; Figures 4C,F–G). Both slices showed eight cambial rings, the two outermost barely visible by the bright lines of the cambia; the parenchyma in the upper slice (Figures 4B,C) appeared much darker than the cambia and only structured by radial lines whereas, in the lower plane (Figures 4D,E), the parenchyma was much brighter in the middle part and almost as bright as the cambia; the core of the beet had a diffuse split shape in the upper plane (Figure 4C), while in the lower plane (Figure 4E), it showed a bright round structure. The onset of side roots disturbing the regular ring structure is clearly visible in Figure 4D on the left side. These anatomical details of the core and the side roots both point to the lower section (Figures 4D,E) belonging to the root part of the beet. The upper section (Figures 4B,C) on the other hand was obviously located in the hypocotyl/shoot part of the beet.

**Figure 5:**
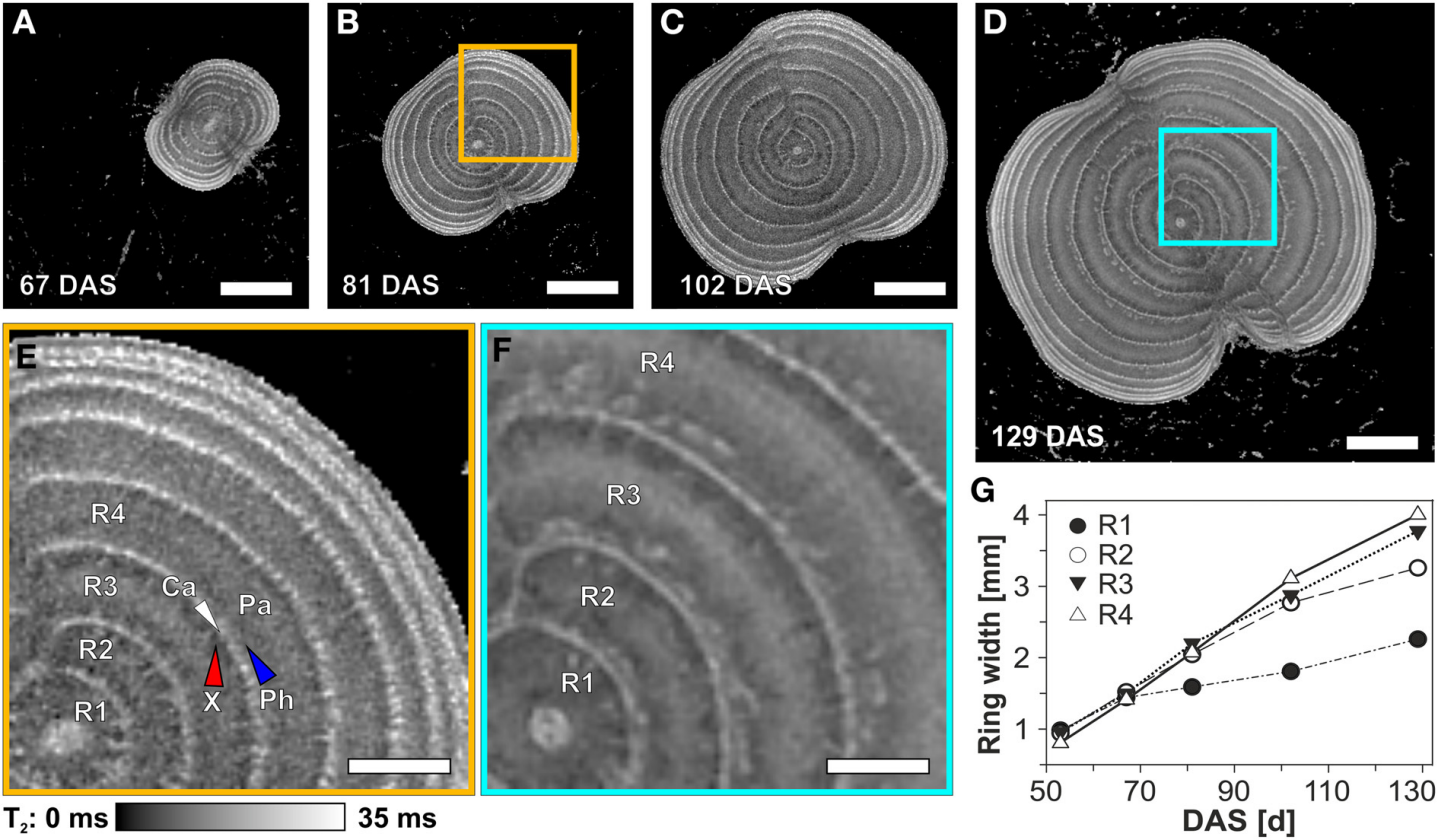
**Development of the rings of a sugar beet at the thickest part of the root region visualized and analyzed by MRI images (T_2_ maps)**. The virtual cross sections **(A–D)** show the development of the beet and the rings between 81 and 129 DAS. Magnified areas at 81 **(B)** and 129 DAS **(D)** are presented in **(E)** and **(F)**, respectively. The development of the width of the innermost four rings over time is shown in **(G)**, including 53 DAS for which the T_2_-map is not shown here. Ring width was measured as the distance between the cambia. For identification of the particular tissues, abbreviations and measurement parameters see Figure 4. Scale bars: **(A–D)** 10 mm; **(E,F)** 5 mm.

From a sugar beet imaged regularly between 53 and 129 DAS, a time series of T_2_ maps of the same location within the beet showed an increasing number of cambial rings during development: 5, 7, 8, and 9 at DAS 67, 81, 103, and 129, respectively (Figures 5A–D). The beet diameter increased by more than a factor of two between DAS 81 and 129, accompanied by a differential broadening of the individual cambial rings. Figures 5E,F, showing magnifications of comparable regions of the beet at DAS 81 and 129, reveals that this increase in distance between cambia appears to be caused by an increase in the width of the parenchyma rings. The width of the inner rings, especially rings two to four, more than tripled, while ring one showed a doubling in width (Figure 5G). During development of the beet, the indentation of the grooves became more pronounced (Figures 5A–D).

## Discussion

### Investigating roots with MRI—sugar beet as a model

Thick roots or other belowground storage organs deliver a large number of agricultural products, such as potato, which ranks as the fourth largest food crop (Viola, 2000), sugar beet, which is the second most important source of sucrose (Biancardi et al., 2010), and ginger, which is used as both a spice and a pharmaceutical resource (Nair, 2013). Nevertheless, application of recently emerging techniques for 3D root imaging in soil has been focused mainly on (“ordinary”) roots of crops such as cereals or legumes with X-ray CT (Mooney et al., 2012) as well as in the first studies involving MRI for investigating 3D root development (Jahnke et al., 2009; Rascher et al., 2011). A similar approach applies for optical imaging of thin roots growing in the artificial environment of transparent gel chambers for crops such as rice (Clark et al., 2011) or barley (Hargreaves et al., 2009). Large size or intricately structured belowground plant organs on the other hand pose special requirements to the imaging methods. This is particularly the case for sugar beet, where the beet shows very complex tissue structures compared to most other storage organs due to its anomalous secondary thickening (Artschwager, 1926), which makes noninvasive assessing of the beet anatomy rather challenging. At later growth stages, the beet develops side roots of increasing size toward its lower end (Artschwager, 1937). This fact can be used to monitor the actual position (alignment) of these side roots in the soil and also to test which sizes of smaller roots can still be visualized with an imaging protocol optimized for a bulk structure. Sugar beet also develops a characteristic morphology and reacts to invasive manipulation with growth disturbances (Artschwager, 1926; Heinisch, 1960), making it an adequate model plant to test the capabilities of monitoring changes of both morphology and anatomy with a non-invasive approach while the beet is growing in the soil.

For MRI measurements of roots growing in soil, some prerequisites must be met concerning pot shape and size as well as substrate properties. Cylindrical containers fit best in the bore of the 4.7 T MRI magnet used in this study. The described mix of agricultural topsoil and coarse sand proved to be well suited for both plant growth and MRI imaging of sugar beets as good image quality was obtained for both the 81 mm I.D. (Figure 4) and the 117 mm I.D. pots (Figures 1, 2). The mineral soil mix was also closer to field conditions than commercial potting soils with often unnaturally high contents of peat. It enabled precise monitoring of belowground plant organs like beets in their native environment including the 3D-alignment of side roots (Figure 1B) not preserved after excavation (Figure 1C). Problems with MRI measurements such as image distortions (Bottomley et al., 1986; Tollner et al., 1991; Heeraman et al., 1997) rendered several authors (Asseng et al., 2000; Mooney et al., 2012) to doubt the usability of MRI for investigating roots in soil in general, however, here we show that by appropriate MRI measurement sequences and settings such artifacts could be minimized. Roots with a diameter down to 500 μm were still detectable with MRI, even in the 117 mm I.D. pots, which fits well with the results where early and therefore relatively simple root systems of maize (Jahnke et al., 2009) or bean (Rascher et al., 2011) were imaged in our lab.

Comparable studies on root systems in soil have been conducted so far mainly with X-ray CT (Mooney et al., 2012; Tracy et al., 2012). This technique can be used to image whole content of pots including soil particles and roots accurately with high spatial resolution for segmenting root systems and their architecture. However, due to the inherently low attenuation contrast levels between roots and soil, rather sophisticated image analysis tools are needed (Mairhofer et al., 2012, 2013; Mooney et al., 2012). MRI offers a much better contrast between roots and soil, which is demonstrated by the fact that the images shown here are only treated by a noise cut-off without any further processing. The requirement to remove strongly ferromagnetic particles from the substrate limits the application of the MRI technique, as soil structure is disturbed after drying and sieving necessary for the mechanized removal. A similar procedure has been reported in high resolution CT studies to get a more homogenous background for better segmenting roots from the soil in the image processing (e.g., Gregory et al., 2003; Hargreaves et al., 2009; Zappala et al., 2013), so this appears to be a common limitation of both techniques. And even such processed soil is much closer to a natural soil compared to other 3D imaging approaches such as gel chambers (Iyer-Pascuzzi et al., 2010). Albeit the soil mix used here worked fine, it remains to be clarified which other soil types can be used. Preliminary experiments in our lab as well as the older survey of a number of American agricultural soil types by Rogers and Bottomley (1987) suggests that several others might be suitable, which would make MRI applicable to a number of different investigations targeting root system architecture and development of other crop species under different soil conditions. Both imaging modalities, MRI and X-ray CT, can be considered to still being at the stage of method development in particular with respect to investigating root biology in the soil. Since the two methods reveal similar but also complementary information, only application studies of root systems of various plants under different conditions will reveal which approach might be best for specific research questions.

As already mentioned we have chosen sugar beet here as a proxy for studying belowground storage organs of plants. For comparison of different plants or genotypes and the detection of treatment effects, the quantification of specific (beet) traits is mandatory. Beet growth (in terms of volume) was non-invasively gathered and the increase in fresh weight over time was approximated. While volume determination with MRI is rather precise due to the high contrast between beet and soil, the accuracy of calculated fresh weight depends on the reliability of beet density taken from reference plants, which were harvested and weighed at similar ages as those in the experiments discussed here, after similar volume determination by MRI. Eventually, the correctness of the calculated fresh weight was verified at the end of an experimental series by measuring the true fresh weight (FW) after harvest (Figure 3). The current study shows that cFW values were a rather good approximation of beet FW in the soil, which allows monitoring the development of individual sugar beet plants over time. The number of samples that can be measured with MRI in studies on morphological development is markedly smaller than in greenhouse trials with pots or rhizotrons. Even automated sample handling systems do not allow measuring more than 20 plants per day at the detail level shown here (Figures 4, 5). On the other hand, this bottleneck might be less relevant due to the fact that trait development monitored on individual plants has less variance compared to measurements by destructive approaches needing different subgroups within a population. And the 3D structure and development of beets and their tissues can be studied only with tomographic techniques providing good contrast and adequate spatial resolution.

### Imaging 3D development of a sugar beet and its potential applications

The distinct shape of a sugar beet and its correlation to sugar storage capability has been studied in the past (De Vries, 1879; Artschwager, 1926; Heinisch, 1960), and researchers had to rely on large numbers of plants and several harvests to get information on average beet development (Ulrich, 1952; Trebbi and Mcgrath, 2009). While different shapes at harvest were extensively characterized and used as criteria in breeding selection, the development of individual beets and its role in performance and yield formation could not be investigated. Here, noninvasive MRI was used to study morphological beet parameters of individual sugar beets such as increase in diameter (Table 1) or different growth rates of both neck and root part (see Figure 2) over time. While at harvest the content of sugar increases from the head to the root region of the beet, parameters lowering the quality for sugar production like potassium, sodium and α-amino N decrease (Mahn et al., 2002), causing different economic values of the beet parts (Draycott, 2006). Therefore, a deeper knowledge of the development of different genotypes may also be valuable for a better understanding of sugar yield. Furthermore, effects of environmental factors (both above- and belowground) on traits of the different beet parts can be studied by repeated imaging. This ability might also prove useful for investigating potatoes, where the morphological traits of the seed tubers affect shoot development in many ways (Struik, 2007). 3D imaging could also be applied for targeted sampling of tissues from actively growing regions of a beet as applied to growing zones of leaves for genetic and metabolic profiling (Matsubara et al., 2006). One important trait of sugar beet visible with MRI early in development is the beet shape, which also affects post-harvest processing. For example soil sticking to the roots in the beet grooves (Figures 2, 5) leads to lesser quality of sugar production and more cleaning efforts are needed (Draycott, 2006).

### Quantification of beet development and effects of container size

Whether pot size affected beet development was monitored by repeatedly measuring individual sugar beet plants with MRI under controlled conditions (Figure 3). Pot size is an important parameter, which, in experimental approaches, should be optimized allowing good plant development while keeping space in a climate chamber and handling efforts within acceptable limits (Poorter et al., 2012b). This is particularly important for MRI experiments where plants and pots also have to fit inside the instrument. In our experiment, beets growing in larger pots showed significantly higher biomass than those in smaller ones already starting at early developmental stages (Figure 3). We measured a significant increase in cFW up to 86% when comparing the plants in the small pots and those growing in the large pots (with a fourfold larger volume). We estimated from this that a doubling of pot size of the smaller pots would have increased the fresh weight by 36% on 118 DAS. This is slightly below the 43% increase of biomass Poorter et al. (2012a) calculated for a doubling of pot volume but within the range of the data cited in the meta-analysis. Obviously the meta-analysis by Poorter et al. (2012a) and our data are not directly comparable since Poorter et al. (2012a) focused on total plant biomass whereas here only the storage organ of sugar beet plants was measured. It shows, however, that also for designing experiments with potted sugar beets and maybe other storage organs, possible differences in developmental stages or accumulation of biomass need to be considered when different pot sizes are used. The statistically significant difference found for the whole growth curves in Figure 3 demonstrates that the pot size effect was already effective at the early stages of beet development when counter-pressure of the pot wall could not have come into play. Also, spacing between pots was large enough to prevent overlap of canopies and water and nutrient supply was similar. Together, this indicates that confinement not of the beets themselves but of the regular roots may have affected the beet fresh weight development as suggested for plant biomass in general by Poorter et al. (2012a). Studies on other root systems may benefit from such dynamic analysis of root biomass and its distribution within the pot for investigating the mechanisms of growth limitation by root confinement. This is a relevant question in many respects as the high throughput systems for automated phenotyping, which have been developed in recent years (Granier et al., 2006; Nagel et al., 2009), have pot size restrictions by necessity and, even in the field, rooting space may be limited e.g., by compacted soil patches (Hatfield, 1992). In addition to the pot size effects shown here on beet development, MRI can be used also to study other stress response dynamics such as effects of pathogen infection on roots or storage organs, which may occur before they become detectable aboveground.

### Tissue identification and mapping

All storage organs are composed of characteristic combinations of tissues to fulfill their biological function, which is mainly storage of carbohydrates, lipids, and/or proteins to support offspring or following year development. At the same time the efficiency of storage defines the commercial yield of crops such as potato, yam, sweet potato, or sugar beet. Concerning the internal structure of a sugar beet, one of the key processes is the development of the rings consisting of cambia, vascular and storage tissues. T_2_ mapping with high spatial resolution allows for detailed investigations since T_2_ is often highly correlated with cell and/or vacuole size (positive correlation; Macfall and Johnson, 1994; Edzes et al., 1998; Van Der Weerd et al., 2001) and membrane permeability (negative correlation; Van Der Weerd et al., 2002; Van As, 2007). Also negatively correlated are the usually weaker effects of cell wall thickness and the concentration of solutes like sugars in the vacuoles (Callaghan et al., 1994; Raffo et al., 2005). A more detailed explanation of the factors influencing T_2_ in plant tissues can be found in Supplementary information 1. The longest T_2_ times were found in the cambia (Figures 4, 5) possibly caused by very thin cell walls typical for meristematic tissues (Esau and Evert, 2006) as validated in light microscopic images (e.g., Figure 4G). Similar long T_2_ times of cambia as compared to the surrounding parenchyma were reported for apple fruits (Sibgatullin et al., 2010). The increasing T_2_ times in the middle of the parenchyma rings over time (compare Figures 5E,F) can be explained by an increase in vacuole size following cell expansion as described for this tissue (Artschwager, 1930; Zamski and Azenkot, 1981). Shorter T_2_ values of xylem and phloem areas may be due to ten times smaller cell diameters for both tissues compared to the cells in the parenchyma rings (Artschwager, 1930; Zamski and Azenkot, 1981). For the xylem, the thick cell walls (Artschwager, 1930) may also play a role in shortening T_2_ time just as the high sugar content of the sieve tubes and the apoplast of the phloem parenchyma in the phloem (Fieuw and Willenbrink, 1990). While a strong T_2_ contrast between the vascular bundles and the surrounding tissues was already reported by Macfall and Johnson (1994) for excavated sugar beets, this study shows that it can be measured also on undisturbed beets while growing in the soil. The MRI T_2_-mapping of tissue structures is still challenging since the correlation of the T_2_ values with actual cell or tissue properties has to be evaluated for each species against light microscopy of particular tissue sections. Nevertheless, the possibility of studying tissue development in storage organs *in vivo* suggests possible applications also on other plants with anomalous cambial development such as sweet potato (Villordon et al., 2009).

Beside the total biomass of sugar beet, a major factor determining sugar yield is the sugar concentration, which is closely tied to the ring structure (Artschwager, 1926; Draycott, 2006). This has been studied extensively in the past (e.g., De Vries, 1879; Artschwager, 1926, 1930; Milford, 1973) to understand the origins of the supernumerary cambia, which form the beet. The rings with their phloem and parenchyma zones are critical for providing transport and sugar storage capacities (Artschwager, 1926; Draycott, 2006). No clear correlation with sugar yield was found for simple traits such as number and width of rings or parenchyma cell size at harvest (Draycott, 2006), even though several studies argued that, with shorter distances between phloem and storage tissues, a higher sucrose content should be achievable (Milford, 1973; Wyse, 1979; Doney et al., 1981). Also cDNA cloning of extracellular and vacuolar sucrose cleaving enzymes revealed a change in the mechanisms of the functional unloading pathways during the first weeks of beet development, and transcript profiles revealed developmental and metabolic changes at similar or later age (Godt and Roitsch, 2006; Bellin et al., 2007; Trebbi and Mcgrath, 2009). However, it could not be correlated with the development of structural traits such as tissue volume or growth rates. This lack of understanding may be one reason why the total sugar content of commercial sugar beet lines has not been risen significantly in the last decades (Draycott, 2006). The application of T_2_ mapping on sugar beets offers a way to non-invasively identify different tissues, and to trace and analyze the development of the tissues over time. This approach will allow investigating mechanisms of structural and functional development of sugar beets for example by investigating different genotypes with contrasting development patterns and also by correlating the MRI-data on temporal tissue development with transcript and metabolic profiles at different time points. Noninvasive methods like MRI may thus contribute to a better understanding of possible correlations between tissue development of storage organs and final yield of quality compounds ranging from sugar in sugar beet or starch in potato to specific drug precursors as in red beet or ginseng.

## Author contributions

The concept of the method and instruments was elaborated by Dagmar van Dusschoten, Ulrich Schurr, and Siegfried Jahnke. Ralf Metzner, Dagmar van Dusschoten and Siegfried Jahnke did the design of the study and development of experimental protocols. Experimental work and data acquisition was done by Ralf Metzner. Data analysis was by Ralf Metzner and Jonas Bühler while interpretation was by Siegfried Jahnke and Ralf Metzner. Ralf Metzner performed the drafting of the manuscript with accompanying critical input from Jonas Bühler, Siegfried Jahnke, Dagmar van Dusschoten and Ulrich Schurr. All authors approved the final version and declare to be accountable for all aspects of the work published.

### Conflict of interest statement

The authors declare that the research was conducted in the absence of any commercial or financial relationships that could be construed as a potential conflict of interest.
