# Supplementary material for: Belowground plant development measured with magnetic resonance imaging (MRI): exploiting the potential for non-invasive trait quantification using sugar beet as a proxy
Source: Front Plant Sci. 2014 Sep 16;5:469. doi: 10.3389/fpls.2014.00469 (PMC4165221; doi:10.3389/fpls.2014.00469)
Supplement: Supplementary file 2 [file DataSheet1.DOCX]

Frontiers in Plant Sciences Supplementary Material

Technical Advances in Plant Sciences 02.07.2014

*Supplementary Material*

Belowground plant development meets magnetic resonance imaging (MRI): exploiting the potential for non-invasive trait quantification using sugar beet as a proxy

**Ralf Metzner*, Dagmar van Dusschoten, Jonas Bühler, Ulrich Schurr and Siegfried Jahnke**

Institute of Bio- and Geosciences, IBG-2: Plant Sciences, Forschungszentrum Jülich GmbH,

Jülich, Germany

***Correspondence:**Dr. Ralf Metzner, Institute of Bio- and Geosciences, IBG-2: Plant Sciences, Forschungszentrum Jülich GmbH, 52425 Jülich, Germany

Email: r.metzner@fz-juelich.de

**Supplementary information 1**

**Correlation between T_2_ contrast and certain tissue structures**

Detailed explanations of the physico-chemical processes that are the basis of correlations between T_2_ times values and specific tissue structures are described in the literature ([Veres et al., 1991](#_ENREF_8);[Edzes et al., 1998](#_ENREF_3);[van der Weerd et al., 2002](#_ENREF_7)), but with respect to the importance for interpreting MRI images for *in-vivo* histology of plant tissue we will give a brief summary.

Solid matter has in general an extremely short T_2_ (<20µs) as compared to pure water (2s) and therefore will not give a signal itself with our measurement settings. However, due to the fast exchange of excited water protons with OH^-^, e.g. of cellulose, the presence of solid matter will significantly shorten the T_2_ of the water in its vicinity ([Ibbett et al., 2008](#_ENREF_4)).Therefore any water protons able to exchange with the cell wall cellulose will result in short T_2_ times. If this has an effect on the T_2_ times in the respective pixel depends on the fraction of water interacting with the cell walls. If water can exchange with the walls depends – at a time frame given by the experimental settings - mainly on two tissue properties (1) cell (and/or vacuole) diameter and (2) membrane permeability. The smaller the diameter of the cells and the higher the permeability of tonoplast and plasma membrane the higher is the probability of water protons to reach the wall and interact. Therefore both smaller cell size and higher membrane permeability will result in short T_2_ times compared to larger cells and lower membrane permeability.

Overall T_2_ times of sugar beets in our measurement were much shorter compared to those found for other plant tissues (e.g. [Veres *et al.*, 1991](#_ENREF_7); [Robinson *et al.*, 2000](#_ENREF_5)). One possible reason would be strong differences in cell size between sugar beets and the plants used in these studies. While this cannot be ruled out completely due to the diversity of cell sizes among plant tissues judging from the light microscopic images published there the cell diameters appeared comparable to sugar beet. Therefore it appear likely that a high membrane permeability is the reason for those short T_2_ times, which also correlates well with the findings of [Alleva et al. (2006](#_ENREF_1)) that sugar beet root tissues have much higher membrane permeability for water than plasma membranes in other plants.

Another factor that has been observed frequently that shortens T_2_ times for water protons are air pockets in plant tissues causing magnetic susceptibility contrast ([Veres et al., 1991](#_ENREF_8);[Duce et al., 1992](#_ENREF_2)). However, for sugar beet this factor appears to be negligible as the contrast parameter T_1_ in not influenced by susceptibility in contrast to T_2_ but the map of T_1_ showed the same contrast pattern as the T_2_ maps (data not shown). Furthermore, when other factors influencing T_2_ such as cell size are mapped with independent methods, in high sugar containing tissues T_2_ times can even be used to conclude on sugar content (e.g. [Verscht *et al.*, 1998](#_ENREF_48); Melkus et al, 2009), so MRI can potentially even be used for mapping and quantification of sugar distribution in sugar beets *in vivo*.

Alleva, K., Niemietz, C.M., Maurel, C., Parisi, M., Tyerman, S.D., and Amodeo, G. (2006). Plasma membrane of Beta vulgaris storage root shows high water channel activity regulated by cytoplasmic pH and a dual range of calcium concentrations. *Journal of Experimental Botany* 57**,** 609-621. doi: 10.1093/jxb/erj046.

Duce, S.L., Carpenter, T.A., Hall, L.D., and Hills, B.P. (1992). An investigation of the origins of contrast in NMR spin echo images of plant tissue *Magnetic Resonance Imaging* 10**,** 289-297. doi: 10.1016/0730-725x(92)90488-l.

Edzes, H.T., Van Dusschoten, D., and Van As, H. (1998). Quantitative T-2 imaging of plant tissues by means of multi-echo MRI microscopy. *Magnetic Resonance Imaging* 16**,** 185-196.

Ibbett, R.N., Schuster, K.C., and Fasching, M. (2008). The study of water behaviour in regenerated cellulosic fibres by low-resolution proton NMR. *Polymer* 49**,** 5013-5022. doi: 10.1016/j.polymer.2008.08.053.

Melkus, G., Rolletschek, H., Radchuk, R., Fuchs, J., Rutten, T., Wobus, U., et al. (2009). The Metabolic Role of the Legume Endosperm: A Noninvasive Imaging Study. *Plant Physiology* 151**,** 1139-1154. doi: 10.1104/pp.109.143974.

Robinson, A., Clark, C.J., and Clemens, J. (2000). Using H-1 magnetic resonance imaging and complementary analytical techniques to characterize developmental changes in the *Zantedeschia* Spreng. tuber. *Journal of Experimental Botany* 51**,** 2009-2020.

Van Der Weerd, L., Melnikov, S.M., Vergeldt, F.J., Novikov, E.G., and Van As, H. (2002). Modelling of self-diffusion and relaxation time NMR in multicompartment systems with cylindrical geometry. *Journal of Magnetic Resonance* 156**,** 213-221. doi: 10.1006/jmre.2002.2550.

Veres, J.S., Cofer, G.P., and Johnson, G.A. (1991). Distinguishing plant tissues with magnetic resonance microscopy. *American Journal of Botany* 78**,** 1704-1711.

Verscht, J., Kalusche, B., Kohler, J., Kockenberger, W., Metzler, A., Haase, A., et al. (1998). The kinetics of sucrose concentration in the phloem of individual vascular bundles of the Ricinus communis seedling measured by nuclear magnetic resonance microimaging. *Planta* 205**,** 132-139. doi: 10.1007/s004250050304.
